# A20 upregulation during treated HIV disease is associated with intestinal epithelial cell recovery and function

**DOI:** 10.1371/journal.ppat.1006806

**Published:** 2018-03-05

**Authors:** Avantika S. Chitre, Michael G. Kattah, Yenny Y. Rosli, Montha Pao, Monika Deswal, Steven G. Deeks, Peter W. Hunt, Mohamed Abdel-Mohsen, Luis J. Montaner, Charles C. Kim, Averil Ma, Ma Somsouk, Joseph M. McCune

**Affiliations:** 1 Division of Experimental Medicine, University of California, San Francisco, San Francisco, CA, United States of America; 2 Division of Gastroenterology, University of California, San Francisco, San Francisco, CA, United States of America; 3 Division of HIV/AIDS, University of California, San Francisco, San Francisco, CA, United States of America; 4 The Wistar Institute, Philadelphia, PA, United States of America; Vaccine Research Center, UNITED STATES

## Abstract

Untreated Human Immunodeficiency Virus (HIV) infection is characterized by intestinal epithelial barrier dysfunction and chronic inflammation, related features that are attenuated to variable degrees by suppressive antiretroviral therapy (ART). Specific mediators of intestinal epithelial cell (IEC) dysfunction and restoration during HIV disease and treatment have yet to be identified. We studied IECs isolated from intestinal biopsies by RNAseq and found that mRNA levels for the ubiquitin-modifying enzyme, A20, are upregulated in ART-treated individuals and are positively correlated with markers of epithelial function (e.g., *CTNNB*, *CLDN4*, and *TJP1*). In a murine intestinal organoid model, A20 expression was suppressed by interferon-alpha (IFNα), which is highly expressed during HIV viremia and induces IFN-mediated signaling. Notably, A20 deletion rendered intestinal organoids more susceptible to cell death and inhibition of barrier-related genes mediated by interferon-gamma (IFNγ), a cytokine also present at elevated levels during untreated infection. Furthermore, A20 specifically restricted expression of IL-17A-induced inflammatory genes in organoids. Finally, ART-suppressed chronically infected individuals treated with pegylated IFNα2a for five weeks demonstrated reduced expression of A20 in peripheral blood mononuclear cells. Our results are thus consistent with a model in which enhanced type I interferons suppress A20 levels, leading to IFNγ-mediated dysfunction. As such, variation in A20 expression during the course of HIV infection could underlie both the development of epithelial dysfunction before the initiation of ART and the recovery of intestinal epithelial integrity thereafter.

**Trial registration:**

ClinicalTrials.gov Clinical Trial NCT00594880

## Author summary

Though the advent of antiretroviral therapy (ART) has significantly improved the lives of individuals infected with the Human Immunodeficiency Virus (HIV), those on therapy still suffer from an enhanced risk of morbidities and mortalities that is caused, at least in part by, overactivation of the immune system. Disruption in the intestinal epithelial barrier, which when intact cordons off the immune system from components in the gut that can drive an inflammatory response, is thought to contribute significantly to this process. How HIV causes this disruption has not been identified. In this study, we analyzed the expression profile of intestinal epithelial cells from HIV-infected individuals, on and off therapy, compared to uninfected controls and observed an increase in expression of an anti-inflammatory regulator, A20, in treated participants but not in those without treatment. In a culture system that parallels features of the intact gut barrier, we saw that a cytokine induced by HIV suppressed the expression of A20. In addition, treatment of ART-suppressed HIV-infected individuals with this same cytokine led to reductions in A20 expression. Furthermore, experimental deletion of A20 in our culture system and treatment with HIV-associated cytokines led to changes consistent with a defective intestinal barrier. As such, we suggest A20 is central to epithelial dynamics during HIV disease and could protect the epithelium from damage associated with HIV-induced cytokine changes.

## Introduction

Infection with Human Immunodeficiency Virus (HIV) results in widespread inflammation in infected individuals with key changes in the immune system that have important clinical implications. Untreated HIV disease is characterized by high levels of systemic and tissue-localized proinflammatory cytokines such as an increase in type I interferon activity and interferon-gamma (IFNγ), each of which is thought to contribute to tissue damage [1,2]. Importantly, these hallmarks of infection result in immune dysfunction that can often persist despite the initiation of antiretroviral therapy (ART). Immune dysfunction, as measured by type I IFN activity for example, is predictive of non-AIDS related morbidity and mortality in ART-treated individuals [3–6] and residual immune activation may account for the overall shorter life expectancy of HIV-infected subjects, particularly in those with incomplete immunologic restoration after ART initiation [7–12].

HIV infection also causes significant disruption of the mucosal immune system, a site of progressive CD4+ T cell loss and a tissue that harbors a substantial reservoir of latent HIV [13,14]. In particular, progressive SIV/HIV disease is associated with the preferential depletion of CD4+ T helper type 17 (Th17) cells producing the cytokine interleukin 17 (IL-17), a key epithelial support cytokine whose depletion during viremia is proposed to be the mechanistic basis for epithelial barrier dysfunction and pathogenesis [15–19].

Intestinal epithelial cells (IECs) play both physical and physiological roles in maintaining gut homeostasis [20] by acting as a spatial separation between luminal contents and underlying immune cells and by expressing and secreting various molecules that promote barrier integrity. HIV-infected individuals exhibit a defect in the epithelial barrier, as characterized by increased epithelial permeability, decreased expression of tight junction proteins [21–23], and elevated cell death [24] during viremia. ART can reverse these changes, albeit to variable degrees [21,24,25], and it is thought that residual immune alterations during ART may be due to continued permeability of the epithelial barrier, driving a chronic inflammatory response against luminal microbial contents [26]. While epithelial dynamics have been studied at the intestinal level, specific investigation of the epithelial compartment in HIV-infected patient cohorts has not been conducted in a systematic manner. Furthermore, specific mediators of IEC dysfunction and restoration during HIV disease and treatment have yet to be identified.

Given the lack of evidence for direct infection of IECs with HIV *in vivo* [27,28], we hypothesized that the changes in epithelial dynamics during infection are effected indirectly by HIV via virus-induced changes in cytokine secretion, resulting in barrier dysfunction and disease progression. Reciprocally, we speculated that these pathways may also determine the extent of disease resolution after the initiation of ART. To address these hypotheses, we analyzed intestinal epithelial cell isolates and lymphocytes from primary human intestinal tissue obtained from HIV-infected individuals at varying stages of disease and treatment. Here, we show that the induction of the ubiquitin-modifying enzyme, A20, is associated with markers of epithelial function after the initiation of ART. We also demonstrate that deletion of A20 in a mouse intestinal organoid model is associated with hallmarks of epithelial dysfunction, particularly in the presence of IFNγ. On the basis of these findings, we speculate that changes in the expression of A20 may play a pivotal role in the dynamics of the intestinal barrier during untreated and treated HIV disease.

## Results

### Characteristics of the study participants

We studied 34 participants from the SCOPE cohort at Zuckerberg San Francisco General Hospital, which included risk-matched uninfected controls (n = 9), untreated HIV viremic participants (n = 6), and those effectively suppressed on ART (n = 19) (**Table 1**). Intestinal biopsies were obtained by flexible sigmoidoscopy and analyzed. Alterations in immunological measurements were consistent with prior observations [11,15,23]. Compared to HIV-uninfected participants, viremic individuals had a significantly lower proportion of gut CD4+ T cells and a commensurately increased proportion of CD8+ T cells (**S1A Fig**). The frequencies of these cell subpopulations were partially but incompletely restored towards normal in the ART-treated subgroup. The frequencies of gut-associated IL-17A- and IL-22-producing CD4+ T cells were significantly lower in untreated subjects and largely restored to normal levels amongst ART-suppressed participants (**S1B–S1D Fig**). Importantly, the production of IL-17A by all lymphocytes (gated as all CD45+ cells) paralleled that found with CD4+ T cells, indicating broad IL-17A depletion in the gut during viremic HIV (**S1E Fig**). Despite full restoration of the percent of T cells expressing IL-17A and IL-22, signs of peripheral immune activation (as reflected by CD8+ T cells co-expressing CD38 and HLA-DR) remained elevated in ART-treated patients relative to uninfected controls, suggesting persistent activation of the immune system despite viral suppression (**S1F Fig**).

**Table 1:** Clinical characteristics of the cohort.

Subgroup	Uninfected (n = 9)	Viremic Untreated (n = 6)	HIV+ ART+ (n = 19)
**Age**	50 (11)	48 (3)	55 (9)
**Gender**	100% male	100% male	95% male
**CD4 T Cell Count (cells/ml)**	656 (282)	486 (103)	573 (384)
**Nadir CD4 Count (cells/ml)**	NA	410 (94)	150 (236)
**Plasma Viral Load (copies/ml)**	NA	24,869 (23,465)	NA

Measurements of indicated characteristics by clinical subgroup. For all parameters, the median is given, with the interquartile range following in parentheses.

### A20 (*TNFAIP3*) is upregulated in IECs from ART-treated individuals

**Fig 1:**
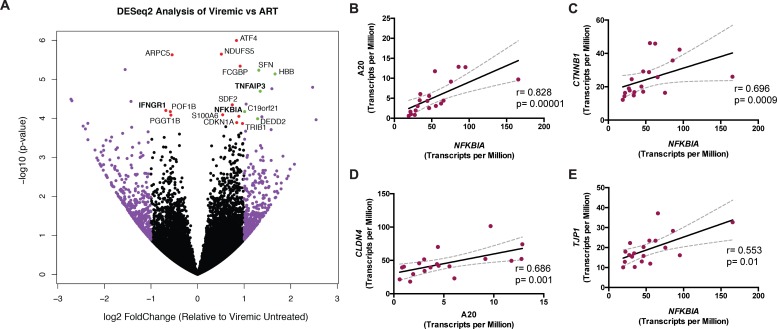
IEC expression pattern from ART-treated participants is consistent with upregulated A20 (*TNFAIP3*) levels. (**A**) Pairwise comparison of IEC gene expression between viremic and ART-treated participants by RNAseq as analyzed by DESeq2 workflow. Each dot represents a sequenced gene. The right side of the plot indicates relative enrichment in ART-treated relative to viremic individuals, whereas the left side indicates the inverse, enrichment in viremics relative to ART-treated participants. Purple signifies greater than two-fold change expression relative to the comparator in either direction. Red indicates a significant p-value (<0.05) after false discovery rate correction. Green indicates fulfillment of both of these criteria. Genes of particular interest are highlighted in bold. (**B**) Spearman correlation of A20 transcript levels and *NFKBIA* expression, as measured by RNAseq, in ART-treated participants (n = 19). Spearman correlation of A20 mRNA or *NFKBIA* gene expression, as indicated on the x-axis, to transcript levels of (**C**) proliferation-associated β-catenin, *CTNNB1*, or the tight junction genes (**D**) *CLDN4* and (**E**) *TJP1*.

To determine which IEC-specific pathways are involved in the observed defects in epithelial function during HIV disease, we utilized a transcriptomics approach to discern gene expression patterns that might be correlated with HIV infection and/or treatment. Whole RNA was obtained from EDTA-isolated IECs from all subgroups and interrogated using RNA sequencing (RNAseq). Pairwise comparisons between IECs from viremic and ART-treated subgroups revealed an expression pattern consistent with upregulated A20 activity during ART (**Fig 1A**). *TNFAIP3*, the gene encoding A20 (and herein referred to as A20), was upregulated in IECs from ART-treated subjects relative to viremic individuals. This was not due to an increased presence of epithelial cells in isolates from ART-treated individuals as the percent of epithelial cells (defined as CD45-EpCAM+) by flow cytometry was similar across subgroups and there was no correlation between transcript levels of A20 and *EPCAM* (**S2A and S2B Fig**). A20 is a known negative regulator of NFκB signaling and plays a key role in restricting inflammatory responses via ubiquitin-editing mechanisms [29–31]. *NFKBIA*, the gene encoding the inhibitor IκBα that directly binds and sequesters NFκB [32], was also upregulated in ART-treated participants relative to viremic individuals and was highly correlated to A20 (p = 0.00001) (**Fig 1B**). Transcript levels of *NFKBIA* were positively associated with those for *CTNNB1*, the gene for proliferation-associated β-catenin (p = 0.0009), suggesting a relationship with epithelial cell survival (**Fig 1C**). Importantly, β-catenin has also been shown to interact with and inhibit NFκB signaling in a manner similar to IκBα [33]. A20 and IκBα (*NFKBIA*) expression also positively correlated with the expression of the tight junction genes *CLDN4* and *TJP1* (p = 0.001 and p = 0.01, respectively) (**Fig 1D and 1E**), consistent with previous work implicating a role for A20 in maintaining gut barrier integrity [34].

A20 transcript levels were also enriched in ART-treated relative to uninfected individuals (**S2C Fig**). Concurrent to A20 upregulation, *IL1R2*, an antagonist of IL-1β signaling [35], was downregulated in ART-treated subjects relative to uninfected individuals. Since IL-1β signaling has been shown to drive A20 expression through NFκB [36], the finding of reduced *IL1R2* is consistent both with previous studies showing elevated IL-1β signaling in SIV-infected monkeys [37] and with the observed upregulation of A20 in ART-treated subjects. RNAseq expression values were validated by targeted qPCR on epithelial isolates (**S2D Fig**). There was no association between A20 levels in treated individuals and ART regimens that did or did not contain the non-nucleoside reverse transcriptase inhibitor, efavirenz, the nucleotide analog reverse transcriptase inhibitor, tenofovir, or the nucleoside reverse transcriptase inhibitor, abacavir (**S2E Fig**), consistent with the hypothesis that the observed changes in A20 expression are due to mechanisms distinct from drug effect alone.

### Viremia-associated type I IFN suppresses A20 levels in intestinal organoids

Despite the fact that A20 expression was upregulated in ART-treated versus viremic individuals (**Fig 1A**), there was no difference in A20 expression between uninfected and viremic subjects (**S2F Fig**). This was the case even though cytokines such as IL-1β and TNF are known to induce A20 [29,36,38] and also known to be elevated in untreated HIV disease [37,39]. Since IFNα has been shown to suppress A20 in dendritic cells in the context of HCV infection [40], we hypothesized that the lack of A20 upregulation during untreated disease might be due to the high levels of type I interferon in viremic participants. Consistent with prior literature [1,17,41], elevated type I interferon activity was observed during untreated HIV, including an increase in the plasma ratio of kynurenine-to-tryptophan (K/T) [3] (**S3A Fig**) and elevated levels of IFN-stimulated genes (e.g., *OAS2* in a statistically significant manner and a trend for *MX1)* in whole gut biopsies (**S3B Fig**).

**Fig 2:**
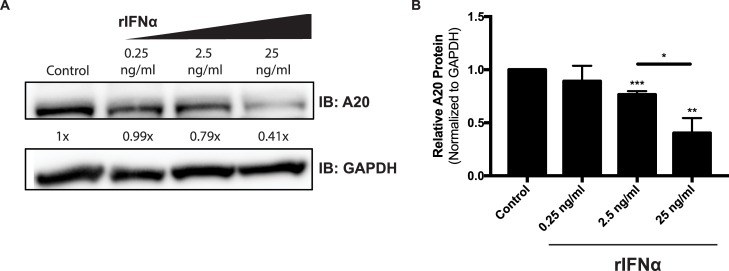
**Intestinal epithelial A20 is downregulated after exposure to IFN**α (**A**) Protein levels of A20, measured by western blot, in murine organoids after treatment with indicated dose of IFNα for 48 hours. (**B**) Quantification of A20 levels normalized to GAPDH in three independent experiments conducted as in (a). Statistical significance was assessed using a t-test. * P<0.05, **P≤0.01, ***P≤0.001.

To determine whether such elevated levels of type I IFN might have a direct effect on A20 expression in IECs, we generated organoid cultures from murine small intestine. Derived from primary tissue, comprised solely of epithelial cells, and exhibiting a crypt/villus architecture, intestinal organoids have been previously shown to serve as a relevant and experimentally tractable model of the intestinal epithelium [42–44]. Organoids were treated with recombinant IFNα for 48 hours and then harvested to measure A20 protein levels by western blot. After incubation with IFNα, A20 levels were found to decrease in a dose-dependent manner (**Fig 2A and 2B**). These observations are consistent with the possibility that A20 is downregulated in viremic individuals by high levels of type I IFN activity.

### A20 downregulation renders epithelial organoids susceptible to IFNγ-mediated reduction of genes that contribute to barrier function

We hypothesized that type I IFN-mediated downregulation of A20 in viremic individuals may render the epithelium more vulnerable to the damaging effects of other proinflammatory cytokines, e.g., IFNγ, that are abundant in untreated disease. As shown in prior studies [45], elevated levels of IFNγ were found in viremic participants at the protein level in terms of frequency of CD8+ T cells producing the cytokine (**S4A Fig**) and at the transcript level in whole rectosigmoid biopsies (**S4B Fig**). Additionally, RNAseq analysis on IECs revealed that *IFNGR1*, which encodes the primary signaling receptor for IFNγ, was upregulated in viremic subjects relative to ART-treated individuals (**Fig 1A**). Taken together, these data suggest that IECs experience higher levels of IFNγ signaling in the viremic state.

**Fig 3:**
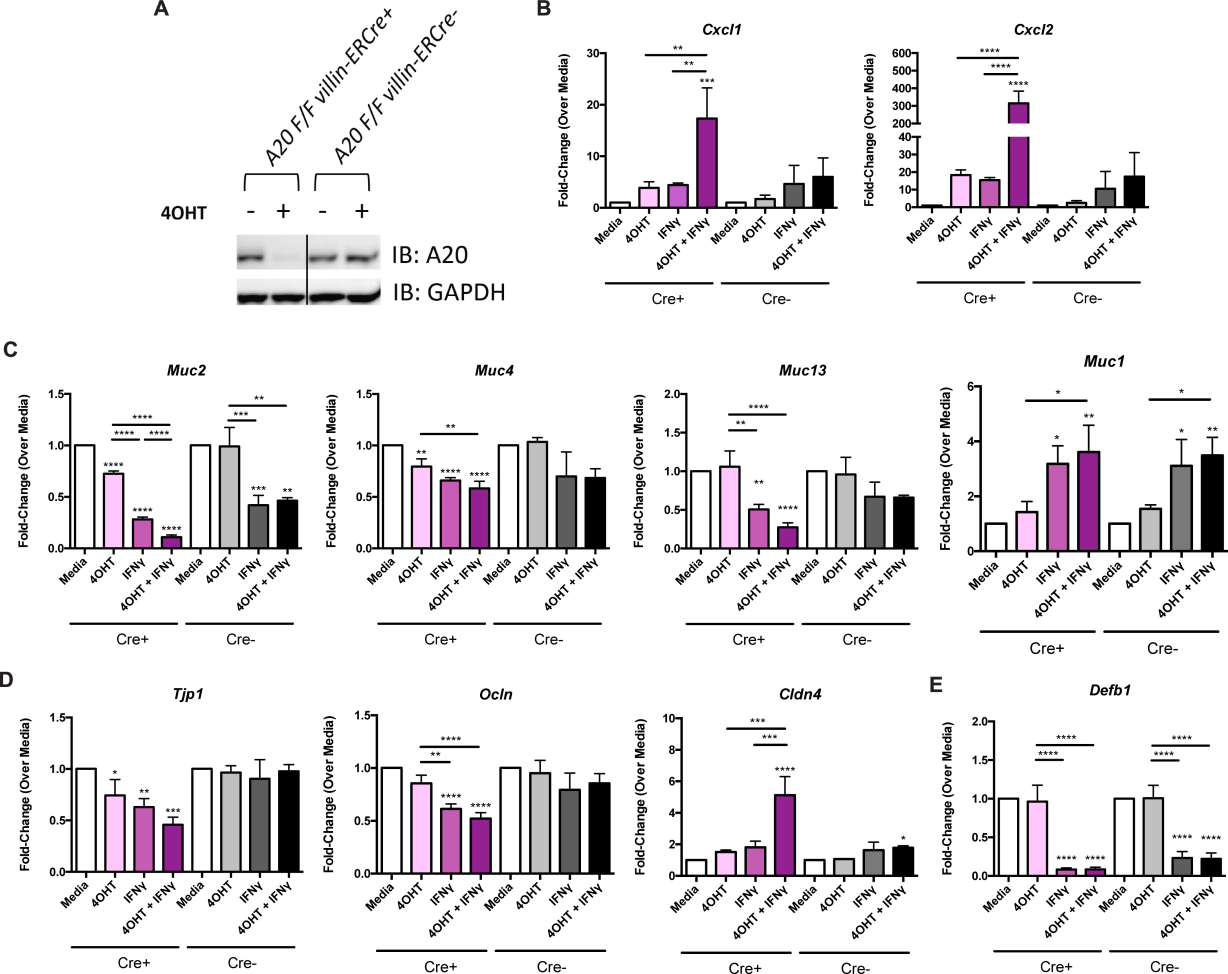
A20 deletion is associated with enhanced IFNγ-mediated inhibition of tight junction and mucin gene expression. (**A**) Western blot for A20 protein levels in A20^FL/FL^ villin-ERCre+ or Cre- organoids after treatment with 4-hydroxytamoxifen (4OHT) for 24 hours. Gene expression by qPCR of (**B**) the proinflammatory chemokines *Cxcl1* and *Cxcl2*, (**C**) the mucins *Muc2*, *Muc4*, *Muc13*, and *Muc1*, (**D**) the tight junction proteins *Tjp1*, *Ocln*, and *Cldn4*, and (**E)** the antimicrobial peptide *Defb1* after stimulation with IFNγ for 12 hours. Statistical significance within Cre+ and Cre- conditions was first tested by ANOVA and specific pairwise comparisons were made using t-tests corrected for multiple comparisons by the Scheffe method (see also S1 Table). Stars above each column indicate statistical significance relative to the media condition. Bars with stars indicate statistically significant differences between the two indicated conditions. * P<0.05, **P≤0.01, ***P≤0.001, ****P≤0.0001.

To test the effect of high IFNγ levels on the expression of genes important to epithelial cell function in the presence or absence of A20, we utilized murine epithelial organoid cultures derived from mice genetically modified to conditionally delete A20. A20^FL/FL^ villin-ERCre^+^ mice are engineered such that, in an epithelial-specific manner (driven by the villin promoter), ligands that signal through the estrogen receptor (ER) induce deletion of A20. 4-hydroxytamoxifen (4OHT), the active metabolite of tamoxifen, is used in culture to quickly and potently induce ER-signaling [46]. As such, A20 deletion in organoids is induced upon 4OHT administration *in vitro*, whereas organoids derived from A20^FL/FL^ villin-ERCre^-^ mice continue to express A20 even after 4OHT administration. Organoids were exposed to 4OHT for 24 hours to allow for A20 deletion, as confirmed by western blot analysis (**Fig 3A**), and then stimulated with rIFNγ for 12 hours, after which transcript levels of genes for inflammatory chemokines, mucins, tight junction proteins, and antimicrobial peptides were assessed (**Fig 3B–3E** and **S1 Table**).

In organoids from A20^FL/FL^ villin-ERCre^+^ mice, A20 deletion alone in organoids, in the absence of exogenous stimuli, had effects on gene expression of a small number of genes, with statistically significant reductions in the transcript levels of the mucin genes, *Muc2* and *Muc4* (**Fig 3C**), and the tight junction gene *Tjp1* (**Fig 3D**). Separately, IFNγ treatment alone led to more widespread changes in gene expression, with significant decreases in transcript levels of mucin genes (*Muc2*, *Muc4*, *Muc13*) (**Fig 3C**) and tight junction genes (*Tjp1*, *Ocln*) (**Fig 3D**). Importantly, when A20 deletion was combined with IFNγ, we observed a robust increase in the expression of the inflammatory chemokines, *Cxcl1* and *Cxcl2 (*15- and 300-fold, respectively) (**Fig 3B**) and further reductions in the expression of *Muc2* transcripts (**Fig 3C**), suggesting an A20-specific role in protecting organoids from IFNγ-mediated inhibition of mucin expression. Similarly, downward trends in the transcript levels for *Muc4*, *Muc13*, *Tjp1*, *and Ocln*, (**Fig 3C and 3D**) indicate that A20 deletion further potentiates the effect of IFNγ, although those changes did not reach statistical significance. These data, in sum, suggest that A20 expression has the capacity to protect IECs from the detrimental effects of IFNγ.

Interestingly, and in contrast to the expression of other measured mucin genes, *Muc1* transcripts increased with IFNγ but were not affected further by deletion of A20 (**Fig 3C**). Previous literature has linked *Muc1* to intestinal inflammation with a specific role in promoting cytokine-mediated immune responses [47], suggesting this might be a mechanism through which IFNγ is exerting its negative effects on the epithelium. In a similar manner, the expression levels of *Cldn4* increased upon IFNγ stimulation and were even further increased upon deletion of A20 (**Fig 3D**). Since *Cldn4* expression has been shown to be directly induced by NFκB [48], the observed increase of this particular gene could be a by-product of ongoing NFκB-related inflammation in the absence of A20. Intriguingly, antimicrobial peptide *Defb1* was markedly inhibited by IFNγ and was not further affected by A20 deletion (**Fig 3E**), suggesting that A20 is not directly involved in regulation of *Defb1* expression but that IFNγ alone can modulate the antibacterial defense of the epithelium.

### A20 downregulation enhances the inflammatory effects of IL-17A

**Fig 4:**
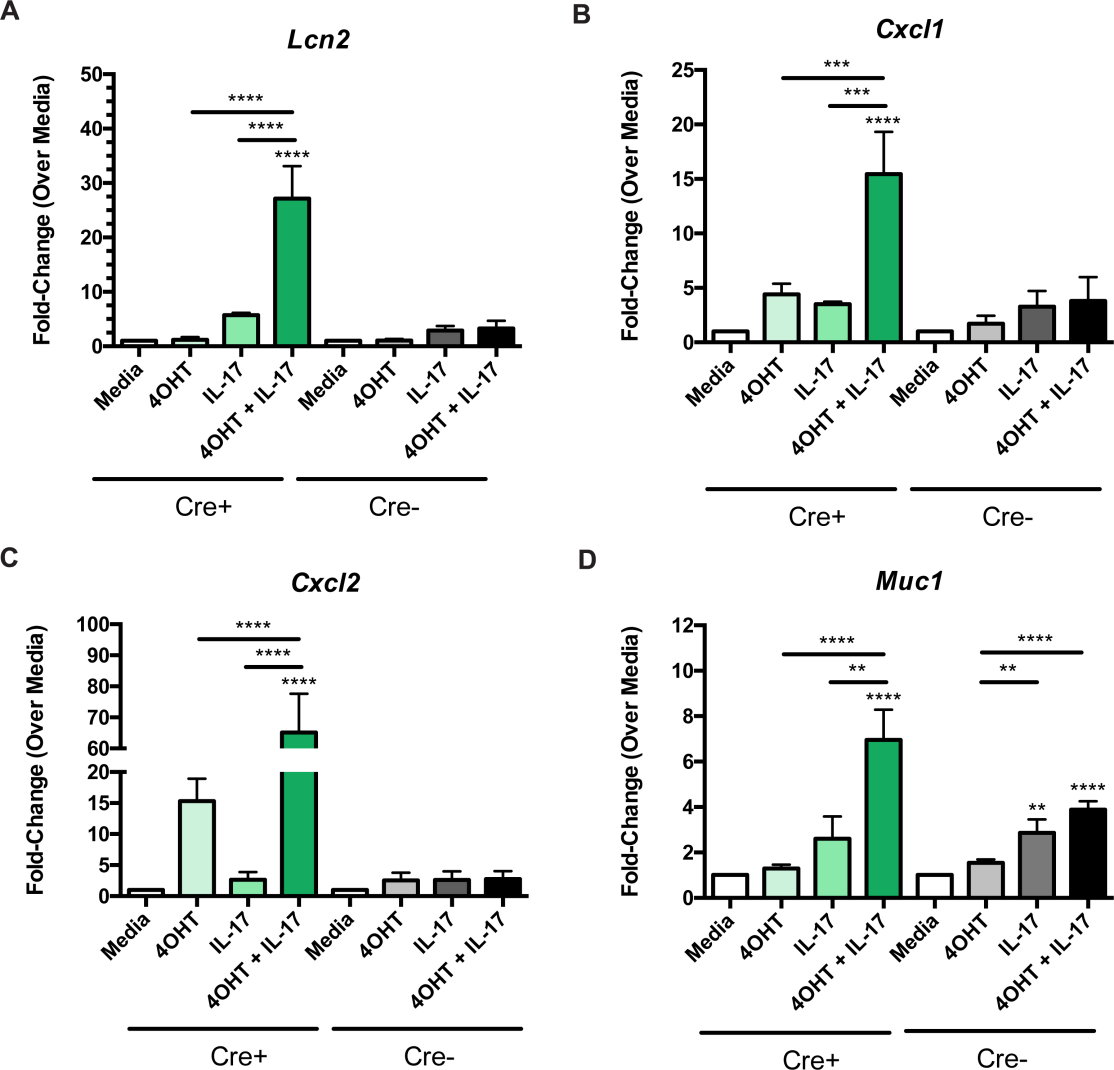
Deletion of A20 enhances the proinflammatory effects of IL-17A. Gene expression of (**A**) *Lcn2*, (**B**) *Cxcl1*, (**C**) *Cxcl2*, (**D**) *Muc1* by qPCR of organoid cultures after 24 hours of 4OHT treatment and/or a 12 hour stimulation with recombinant IL-17A. Cre+ or Cre- conditions were tested separately by ANOVA. Pairwise comparisons were completed post-hoc using t-tests and correcting for multiple comparisons by the Scheffe method (see also S2 Table). Stars above each column indicate statistical significance relative to the media condition of the same organoid line (Cre^+^ or Cre^-^) by a t-test. Bars with stars indicate statistically significant differences between the two indicated conditions. **P≤0.01, ***P≤0.001, ****P≤0.0001.

Given the observation that IL-17A levels are restored to normal in ART-treated subjects (**S1B and S1C Fig**) and prior work in stromal cell lines showed that IL-17 signaling is restricted by A20 [49], we next tested whether A20 deletion altered the effects of IL-17A stimulation in organoid IECs. Intestinal organoids were treated with 4OHT to delete A20 and were then stimulated with rIL-17A for 12 hours. IL-17A stimulation in the setting of A20 deletion led selectively to dramatic increases in the expression of the proinflammatory genes *Lcn2* [50] (by 30-fold), *Cxcl1* (by 15-fold), and *Cxcl2* (by 60-fold) (**Fig 4A–4C** and **S2 Table**). A20-sufficient Cre^-^ organoids showed no such increase in gene expression, suggesting that these changes occur specifically in the absence of A20. Similar to the effect seen with IFNγ, the expression of the mucin gene *Muc1* also increased with IL-17A independent of A20 function (since this effect was also observed in Cre- organoids), with the magnitude of the effect being much more modest than that observed with the inflammatory genes (**Fig 4D**). Taken together, these results indicate that A20 expression in IECs restricts the ability of IL-17 to upregulate pro-inflammatory genes while not affecting the expression of epithelial integrity-related genes (e.g., *Cldn4*, *Tjp1*, *Ocln*, *Muc2*, *Muc 3*, *Muc4*, and *Muc13*) (**S3 Table**).

### Deletion of A20 enhances cytotoxicity induced by IFNα and IFNγ

**Fig 5:**
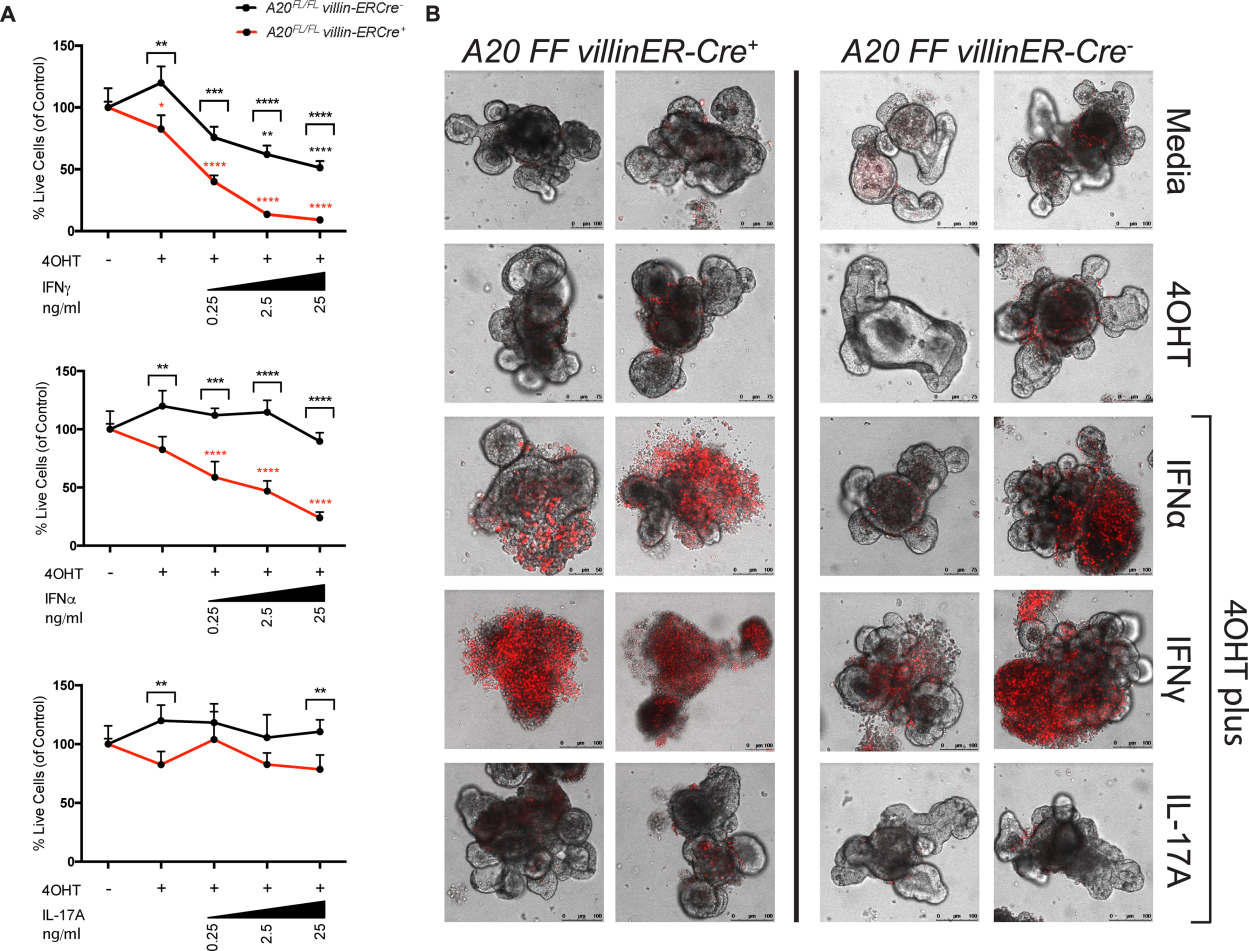
A20 deletion enhances the cytotoxic effects of IFNα and IFNγ. (**A**) Quantification of cell viability by CellTiter Glo 3D assay in organoid cultures after stimulation with three doses of rIFNγ, rIFNα, or rIL-17A for 24 hours. Significance of Cre+ or Cre- was assessed separately by ANOVA. Stars above data points represent statistical significance of cytokine treatment relative to the media control within either the Cre+ (red) or Cre- (black) line after post-hoc comparisons were made by t-test, correcting for multiple comparisons by Scheffe method. Stars above brackets indicate a significant difference between A20-sufficient (Cre-, black) and A20-deficient (Cre+, red) culture within the indicated media condition by t-test. *P<0.05, **P≤0.01, ***P≤0.001, ****P≤0.0001. (**B**) Representative confocal images of propidium iodide (PI)-stained organoids treated for 24 hours with 4OHT followed by 10 ng/ml of indicated cytokine for 24 hours. Two organoids representing the range of PI staining are shown for each condition.

In addition to the effects described above, both IFNα and IFNγ were found to have significant cytotoxic effects on organoid cultures (**Fig 5A and 5B**). Cell death was defined by organoid morphology, propidium iodide staining, and a quantitative ATP-based luminescence assay. Compared with A20-sufficient organoids in media alone, A20-deficient (4OHT-treated) organoids demonstrated a small but statistically significant increase in cell death (**Fig 5A**). When A20-sufficient organoids were treated with the indicated cytokines (IFNγ, IFNα, or IL-17A) (black line in **Fig 5A**), only treatment with IFNγ caused elevated cell death. Treatment of A20-deficient organoids with either IFNα or IFNγ revealed increased levels of cytotoxicity relative to A20-sufficient organoids, an effect that was enhanced upon exposure to increasing concentrations of cytokine (red line in **Fig 5A**). Furthermore, confocal microscopy revealed that A20 deletion combined with IFNγ treatment resulted in a loss of crypt structure in organoids (**Fig 5B**). This is consistent with prior work demonstrating IFNγ induction of epithelial cell death such that IFNγ-knockout animals were protected from epithelial damage *in vivo* in a DSS-colitis model [51,52] and, in epithelial cell lines, IFNγ inhibited epithelial proliferation by suppressing β-catenin activity [53]. In contrast to the phenomena observed with IFNα and IFNγ, IL-17A showed no such effect on cell viability (**Fig 5A and 5B**). These results, together with the observed IFNγ-mediated suppression of epithelial genes (**Fig 3**), suggest that IFNγ may be a primary driver of IEC dysfunction in HIV, an effect exacerbated by type I IFN-mediated downregulation of A20 expression.

### Treatment of HIV-infected ART-treated individuals with IFNα reduces A20 expression *in vivo*

**Fig 6:**
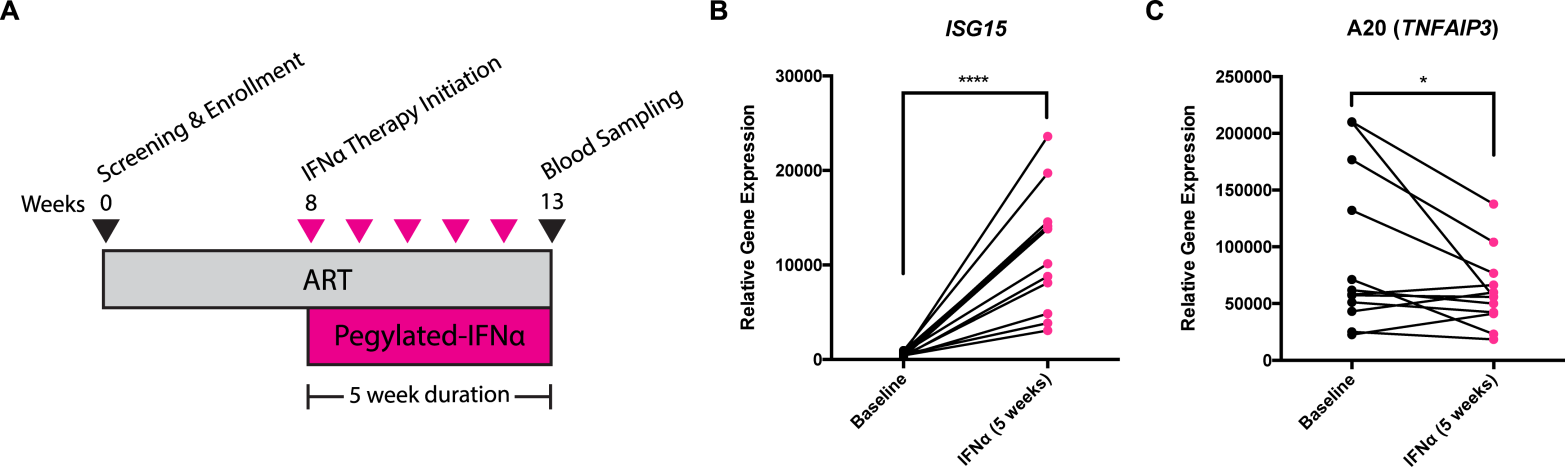
Five week pegylated-IFNα2a immunotherapy of ART-suppressed HIV-infected individuals leads to reduction in A20 expression. (**A**) Schematic showing duration and frequency of pegylated-IFNα2a administration in participants. Transcript levels of (**B**) IFN-stimulated gene ISG15 and (**C**) A20 in PBMCs at baseline and after five weeks of IFNα treatment as assessed by qPCR. Statistical significance was determined by paired t-test. * P<0.05, ****P≤0.0001.

To extend our findings in the organoid system to more clinically relevant scenarios, we assessed the effect of pegylated IFNα2a (Peg-IFNα2a) immunotherapy in ART-suppressed chronically infected subjects on A20 gene expression in samples obtained from a previously published study [54]. Briefly, HIV-infected individuals on ART were treated weekly with Peg-IFNα2a for five weeks, with blood samples taken at baseline and after the immunotherapy period (**Fig 6A**). RNA was isolated from peripheral blood mononuclear cells (PBMCs), and probed for the transcript levels of A20 and a key interferon-stimulated gene, ISG15 [55]. After five weeks of Peg-IFNα2a, the expression of ISG15 was highly elevated in PBMCs, at an average of 21-fold increased from baseline levels (range of 7 to 44-fold) (P≤0.0001) (**Fig 6B**). Concurrently, there was a significant decrease (a mean of 1.56-fold) in the mRNA expression of A20 (P<0.05) (**Fig 6C**), an effect that appeared most dramatic in those with the highest A20 levels at baseline. These observations provide clear evidence that high levels of type I IFN signaling can regulate A20 expression in the context of HIV infection.

## Discussion

Our study is the first to identify a role for A20 in modulating inflammatory signals and epithelial function in the context of HIV infection and treatment. The work outlined here advances our understanding of the molecular underpinnings of epithelial barrier function as well as its relationship to inflammation during HIV infection. It has long been observed that disruption in epithelial homeostasis is a hallmark of HIV infection [21,22,24,25,56]. Prior work examined whole intestinal tissue, while our approach of isolating IECs allowed us to identify A20 as a key regulator of epithelial cell survival and function. These observations deepen our understanding of HIV pathogenesis and further expand our knowledge about the capacity of A20 to regulate epithelial barrier function during viral infections.

**Fig 7:**
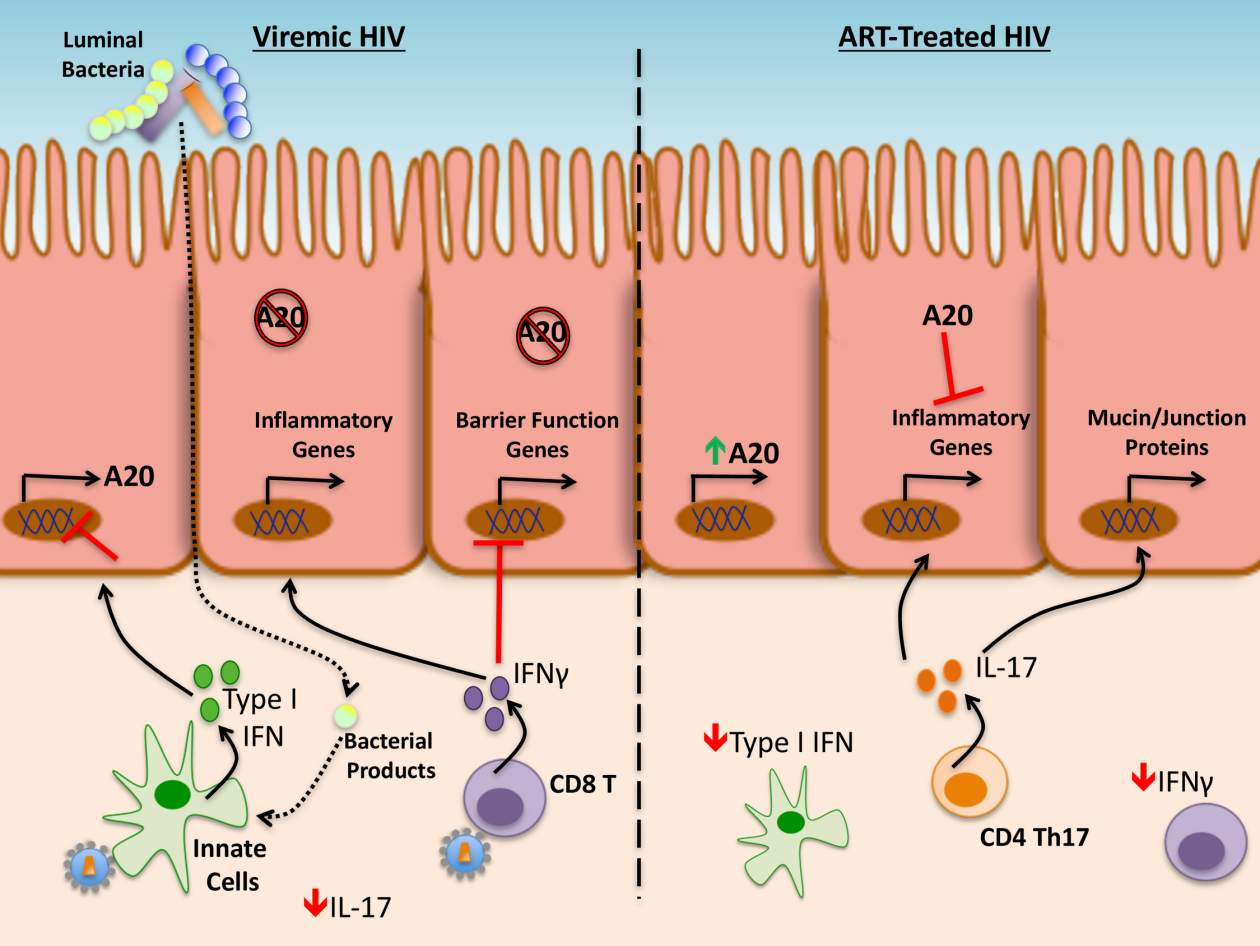
A20 modulation of epithelial function during HIV infection and treatment. Model demonstrating proposed relationship of A20 and relevant cytokines (Type I IFNs, IFNγ, and IL-17A) in the context of untreated (viremic) (left) and ART-treated (right) HIV disease.

Given our findings, we propose that the intestinal epithelial dysfunction found in untreated HIV disease is caused by high levels of type I and type II IFN activity likely mediated in part by IFNα production by plasmacytoid dendritic cells [41,57–59], IFNβ production by TLR4-stimulated macrophages [60], and IFNγ production by CD8 T cells recognizing viral particles respectively [45], that exist concurrently with low levels of IL-17A (**Fig 7**). We show that IFNα is capable of suppressing A20 expression in IECs. Since A20 limits induction of inflammatory genes and cell death by IFNγ, and preserves expression of epithelial function genes (e.g., for some tight junctions proteins and some mucins), high type I IFN levels during untreated HIV disease may render epithelial cells more susceptible to IFNγ-induced damage by preventing A20 upregulation. We speculate that, in the setting of ART, reduction of type-I IFN-mediated signaling [61] results in higher levels of A20, leading to the re-establishment of epithelial homeostasis, as indicated by the positive association between A20 transcript levels and tight junction gene expression in ART-treated individuals. While prior studies defined a role for A20 in maintaining the proper localization of tight junction proteins [34], these results show that A20 can modulate cytokine-mediated inflammatory signaling to promote the production of components associated with epithelial barrier function.

In addition to the impact of the virus itself on the cytokine milieu of the intestine, it is likely that HIV also causes additional shifts in the intestinal immune system that persist in the relative absence of virus, e.g., after the initiation of ART. The intestinal immune system is considered a “trialogue” between epithelial cells, lamina propria immune cells, and the commensal bacteria [62]. Recent work from our lab and others has shown that there is a shift in the composition of microbial communities during HIV infection, the extent of which was associated with inflammatory biomarkers [63,64]. Of note, we found that IFNγ inhibited the expression of a key antimicrobial peptide, *Defb1* (**Fig 3E**). As such, epithelial barrier dysfunction could initially be induced by the combination of proinflammatory cytokines secreted by virus-infected cells, altering the profile of antimicrobial peptides and thus leading to microbial dysbiosis. As productively-infected cells are depleted due to progressive disease and viral particles are no longer the primary driver of inflammation, this dysbiosis appears to result in the translocation of microbial products past the epithelium, resulting in a chronic inflammatory response [26] and pushing forward the cycle of cytokine release and disrupted barrier integrity. The specific stages in which this crosstalk between the epithelium, immune cells, and microbial species occurs remains to be clearly identified, and will be important in understanding the establishment and maintenance of disrupted barrier integrity during HIV infection and treatment.

IL-17A is thought to be important for the maintenance of intestinal mucosal integrity in the case of HIV infection [15–18], yet, it is also capable of driving tissue inflammation [19]. Our data on A20 expression in intestinal organoids suggest these dual effects might be dependent, at least in part, on the presence or absence of A20. Thus, we observe that deletion of A20 clearly enhances the proinflammatory effects of IL-17A signaling (e.g., with increased expression of Lcn2, Cxcl1, and Cxcl2). In the setting of ART, where IL-17A levels rise in the context of ongoing inflammation, this could be particularly important in protecting the epithelium from inflammation-mediated damage. Interestingly, the A20 gene is a susceptibility locus for inflammatory bowel disease (IBD) [65]. IBD is characterized by high levels of IL-17A cytokine [66], yet inhibitors targeting IL-17 pathways have been shown to be ineffective at curbing inflammation [67,68]. Our data suggest that it is the presence or absence of IL-17A coupled with modulation by A20 that is important for disease progression. IL-17A levels did not directly associate with markers of epithelial function in our cohort, as would have been predicted by prior studies [15]; instead, A20 and related genes (e.g., IκBα) demonstrated a positive association with epithelial function. This observation suggests that A20 modulation of cytokine signaling plays an important, and perhaps primary, role in barrier dynamics during HIV disease and treatment.

A caveat attending our studies in intestinal organoids is that the concentrations of IFNα used *in vitro* (0.25–25 ng/ml) are considerably higher than those found in the plasma of acutely SIV-infected rhesus macaques [16,69] or chronically-infected humans [70], in which maximum levels of 0.05 ng/ml have been observed. Nevertheless, a plethora of studies indicate that HIV disease progression is associated with persistent type I IFN signaling [59,71]. Likely, such signaling occurs upon secretion of IFNα and IFNβ (e.g., by plasmacytoid dendritic cells or myeloid cells) within lymphoid or mucosal organs, microenvironments in which type I IFNs levels may be higher than those found in the circulation and also not possible to measure. It will be of interest in future studies to more precisely measure these intra-organ cytokine concentrations and to determine their relevance to the *in vitro* studies presented here.

It is important to recognize that the ART-treated individuals studied here exhibited substantial inter-individual variability in levels of IL-17A, IFNα, IFNγ, and A20. We postulate that such variation, and the balance amongst each of these, might, in turn, reflect differences in the physiologic status of the gut. In addition, though our experiments parsed out the individual effects of IFNα, IFNγ, and IL-17A on epithelial function, these cytokines are expressed concurrently *in vivo*, and their potential interactions were not studied here. In future studies, it will be important to ascertain whether these cytokines potentiate each other (e.g., in the case of IFNγ and IL-17A) or, conversely, if they synergize (e.g., in the case of IFNα and IFNγ) to establish a setpoint of epithelial function.

Treatment of primary organoids with HIV-associated cytokines IFNα and IFNγ, especially when combined with A20 deletion, recapitulated the epithelial biology observed in HIV-infected individuals, specifically in the case of elevating epithelial cell death and inhibiting expression of the tight junction genes, *Ocln* and *Tjp1*. While these data provide clear evidence for A20 downregulation and high proinflammatory cytokine levels as being involved in the establishment of intestinal epithelial dysfunction during HIV, more thorough analyses of tight junction dynamics during the modulation of these pathways will need to be done. Future work should seek mechanistic insight into how A20 is modulated by IFNα and, downstream of this, how IFNα and IFNγ regulate barrier-related genes. Contrary to prior literature that identified a reduction of tight junction expression in viremic subjects [21,22,56] and our findings in organoids demonstrating an impact upon many epithelial function genes, our RNAseq analysis did not reveal differences in tight junctions, mucins, or antimicrobial peptides across participant subgroups. One potential explanation for this discrepancy could be a difference in methodology. While previous studies assessed epithelial function by measuring either transcripts or protein at the whole biopsy level [21,22,56], we specifically isolated and profiled IECs. Thus, observations made in the context of whole biopsies may be more inclusive of events occurring in hematopoietic cells and non-hematopoietic stromal cells. A more robust analysis of genes mentioned above (e.g., tight junctions, mucins, antimicrobial peptides) is warranted, perhaps at the protein level with epithelial specificity, to fully understand the impact of A20 upon epithelial function *in vivo* during HIV infection. It should also be considered that the demographics of the cohort from which we sampled, the SCOPE cohort at UCSF, are such that the vast majority of clinical samples were obtained from male participants [4,17,63]. It is thus important that these analyses be carried out in a cohort more balanced for gender type in future work. Despite these caveats, we were able to reproduce the epithelial barrier dysfunction (e.g., reduced levels of transcripts for tight junction proteins and elevated cell death) observed in prior studies during HIV infection using our organoid model, and expanded upon these findings by identifying key cytokines (IFNα, IFNγ) that are likely to be causing these changes *in vivo*, as well as establishing the importance of A20 levels in mediating these changes. Given prior associations made between A20 and epithelial function [34], the work outlined here indicates potential utility for A20 as a modulator and biomarker of intestinal health in HIV-infected individuals.

In sum, our identification of A20 involvement in epithelial survival and function during HIV infection and treatment is a novel finding that adds to our understanding of intestinal epithelial dysfunction during HIV disease. Further studies are warranted to quantify A20 levels and function in treated HIV-infected individuals, investigating further how A20 modulates epithelial barrier function to affect immune activation and HIV persistence.

## Methods

### Ethics statement

The study from which human tissues were obtained was approved by the institutional review board at UCSF and all participants provided written informed consent (IRB #: 10–01218). All recruited human participants were of adult age and provided consent themselves. Cryopreserved PBMCs were obtained from Clinical Trial NCT00594880. All mice used in this study were housed and bred in a specific pathogen-free facility and euthanized by CO2 inhalation according to the guidelines of the UCSF Animal Care and Use Program (IACUC Protocol #: AN144847). The UCSF Animal Care and Use Program adheres to guidelines established by the Office of Laboratory Animal Welfare (Domestic Assurance #: A3400-01), and is accredited by the Association for Assessment and Accreditation of Laboratory Animal Care (AAALAC) International.

### Study design

The goal of this study was to isolate intestinal epithelial cells in an effort to identify, in a specific manner, how their function changes during HIV infection and treatment. Upon identification of key pathways, we utilized a murine organoid model to determine how manipulation of these pathways altered epithelial function. Detailed information of individual experiments is provided in the sections below.

### Participant recruitment & clinical sampling

HIV-infected individuals and controls were recruited and consented from the SCOPE (Observational Study of the Consequences of the Protease Era) cohort at UCSF for sigmoidoscopy and collecting relevant gastrointestinal biopsy samples for research purposes. HIV-negative controls, HIV viremic untreated, and individuals suppressed with ART were recruited from this cohort. The ART-treated group included HIV-infected individuals maintaining undetectable viral loads (<40 copies/mL) on stable ART for at least one year and spanned a full spectrum of peripheral blood CD4+ T cell recovery, ranging from 220 to 1107 CD4+ T cells per ml of blood. Prior to their procedure, study participants underwent a blood draw and received a Fleets enema. Consenting participants underwent flexible sigmoidoscopy with rectal biopsies obtained at 10–20 cm above the anus using jumbo forceps. Twenty biopsies were placed immediately in 15 ml of RPMI 1640 with 10% fetal calf serum, with piperacillin–tazobactam (500 μg/ml), and amphotericin B (1.25 μg/ml), and transported within one hour to the Division of Experimental Medicine at UCSF, where they were processed the same day. Freshly drawn blood was also transferred to the AIDS Specimen Bank, where plasma was isolated and stored. The study was approved by the institutional review board at UCSF and all participants provided written informed consent (IRB #: 10–01218).

### Cell isolation & processing of primary tissue

Cells of interest were isolated from rectosigmoid biopsies in two sequential ethylenediaminetetraacetic acid (EDTA) (Corning, Fremont, CA) and collagenase (Type II from *Clostridium histolyticum*, Sigma-Aldrich, St. Louis, MO) treatment steps. Briefly, biopsies were washed with phosphate buffered saline (PBS) (Corning) and then incubated in 8 mM EDTA (in Hank’s Buffered Saline Solution) (HyClone, Logan, UT) on a shaker for one hour at 37°C. After vortexing, supernatants were filtered using a 70 μM mesh filter and centrifuged to obtain the IEC fraction. EDTA isolates (estimated to be in the range of 80–90% enriched for IECs, as assessed by flow cytometry) were lysed in Trizol (Thermofisher Scientific, South San Francisco, CA) and RNA was extracted using a phenol-chloroform extraction [72]. RNA Clean & Concentrator 5 (IC) columns (Zymo Research, Tustin, CA) were used to clean RNA preps. Whole tissue RNA was isolated using an AllPrep RNA Isolation Kit (Qiagen, Hilden, Germany). The RNA concentration of all samples was measured on a Nanodrop 1000 Spectrophotometer (ThermoFisher Scientific). Remaining tissue was washed with PBS with 2% FBS and subsequently incubated in 1 mg/ml collagenase in Complete RPMI (RPMI-10 media supplemented with penicillin-streptomycin, L-glutamine, and FBS) for one hour at 37°C on a shaker. Peripheral blood lymphocytes were obtained from whole blood by density centrifugation using Histopaque-1077 (Sigma-Aldrich) and washed in Complete RPMI.

### Flow cytometric analysis of lymphocyte populations

Freshly isolated lymphocytes were analyzed by flow cytometry. Cells were surface stained with Aqua amine reactive dye for viability (Thermofisher Scientific), and antibodies against CD45 (Clone HI30, Alexa 700-Conjugated, Thermofisher Scientific), CD3 (Clone UCHT1, V450-Conjugated, BD Biosciences, San Jose, CA), CD4 (Clone S3.5, PE Texas Red-Conjugated, Thermofisher Scientific), CD8 (Clone 3B5, Qdot 605-Conjugated, Thermofisher Scientific), CD38 (Clone HB7, PE-Conjugated, BD Biosciences), and HLA-DR (Clone L243, FITC-Conjugated, BD Biosciences) for 30 minutes at 4°C, and washed in PBS with 2% FBS. Separately, 5 x 10^5^ cells were plated and stimulated with 10 ng/ml phorbol 12-myristate 13-acetate (PMA) (Sigma-Aldrich) and 1 μg/ml ionomycin (Sigma-Aldrich) at 37°C for five hours in the presence of BD GolgiPlug Protein Transport Inhibitor (BD Biosciences, San Jose, CA). Stimulated cells were surface stained with antibodies against CD45 (Clone H130, PerCPCy5.5-Conjugated, Biolegend, San Diego, CA), CD3 (Clone SP34-2, Pacific Blue-Conjugated, BD Biosciences), CD4 (Clone L200, Brilliant Violet 605-Conjugated, BD Biosciences), and CD8 (Clone 3B5, Qdot 705-Conjugated, Thermofisher Scientific) for 30 minutes at 4°C, fixed in 4% PFA, and then permeabilized in Perm/Wash Buffer (BD Biosciences) according to manufacturer’s instructions. Permeabilized cells were stained intracellularly with antibodies against IL-17A (Clone eBio64DEC17, FITC-Conjugated, eBioscience, San Diego, CA), IL-22 (Clone 142928, PE-Conjugated, R&D Systems, Minneapolis, MN) and IFNγ (Clone B27, Alexa 700-Conjugated, BD Biosciences) and washed in PBS with 2% FBS. Freshly-isolated EDTA-isolates were surface stained with Aqua amine reactive dye for viability (Thermofisher Scientific) and antibodies against CD45 (Clone H130, PerCPCy5.5-Conjugated, Biolegend) and EpCAM (Clone 9C4, BV421-Conjugated, Biolegend). All samples were acquired on a BD LSRII flow cytometer (BD Biosciences) and analyzed using FlowJo 9 software (FlowJo, LLC, Ashland, OR).

### RNAseq & qPCR validation

Samples utilized for RNAseq included epithelial RNA from all participants as well as whole biopsy RNA from a subset of patients, representing each disease subgroup. The whole biopsy samples included five uninfected individuals, all six viremic untreated participants, and five ART-treated individuals selected to span the full range of CD4 count. Total RNA (2 ng) from IEC isolates and from whole rectosigmoid biopsies was converted to pre-amplified cDNA using template-switching reverse transcription via the SMARTer Ultra-Low RNA Input Kit (Clontech, Mountain View, CA), with modified procedures for low input (Fluidigm, South San Francisco, CA) [73,74]. Pre-amplified cDNA libraries were quantified by Quanti-IT PicoGreen dsDNA assay (ThermoFisher Scientific) and normalized to 0.15 ng/ml for input into fragmentation reactions. Fragmentation was performed enzymatically using a Nextera XT DNA kit with indexing primers (Illumina, San Diego, CA). All 50 samples were normalized and multiplexed into a single library. The compiled library was purified by a double-sided bead-based size-selection method using Agencourt AMPure XP beads (Beckman Coulter Genomics, Danvers, MA). A fragment size distribution of 200–500 base pairs for the library was confirmed by running a High Sensitivity dsDNA assay on a Bioanalyzer 2100 (Agilent Technologies, Santa Clara, CA). A quality-controlled library was sequenced as 50-base single end reads on a HiSeq 4000 in rapid-output mode at the UCSF Center for Advanced Technology (San Francisco, CA). Sequence reads were aligned to the human genome (GRCh37) using STAR [75], and gene expression estimation was performed with RSEM [76]. Differential expression analysis was performed on counts data using DESeq2 [77], and candidate genes were false discovery rate corrected prior to determining lists of significantly altered genes. Data were visualized as transcripts per million (TPM). All data are available in the NCBI Gene Expression Omnibus under accession GSE81198.

To validate RNAseq results, original RNA isolates from each sample were used to generate cDNA by reverse transcriptase using the Omniscript RT kit (Qiagen). Amplification was performed on a StepOnePlus (Applied Biosystems, Foster City, CA) with PrimePCR SYBR Green Assays (Bio-Rad Laboratories, Hercules, CA) using the assay-specified cycling protocol. After normalization to housekeeping genes *B2M* and *ACTB*, the fold-change in expression of target genes (*TNFAIP3*, *NFKBIA*, *IL1R2*) was calculated relative to the uninfected subgroup and correlated against fold-change calculations of RNAseq data from matched participants.

### Measurements of plasma markers of inflammation

Concentrations of kynurenine and tryptophan in the plasma were measured by high-performance liquid chromatography–tandem mass spectrometry, as previously described [17].

### Whole biopsy qPCR

To measure transcript levels of cytokines in the gut, qPCR was conducted on whole rectosigmoid biopsy RNA, isolated as above. IFNγ mRNA expression was assessed using a Taqman Gene Expression Assay (Thermofisher Scientific). Expression was normalized to the housekeeping gene *ACTB* and fold-change calculated for each participant relative to the average of all uninfected controls.

### Mice

A20 flox mice were generated in the Ma lab as described previously [78]. Transgenic mice harboring a tamoxifen-inducible Cre recombinase under the control of the villin-promoter (villin-ER-Cre) were a kind gift from S. Robine (Institut Curie-CNRS, Paris, France) These two strains of mice were intercrossed to generate A20^FL/FL^ villin-ERCre^+^ mice, used for the establishment of organoids. All mice used in this study were housed and bred in a specific pathogen-free facility according to the IACUC guidelines of UCSF.

### Organoid culture establishment & maintenance

Murine small intestinal organoids were established as previously described [42], with modifications. Intestinal crypts were isolated from the small intestine and cultured as outlined, with substitution of 10% R-spondin1 conditioned medium for recombinant R-spondin1, and the addition of Normocin (100 mg/ml, Invivogen, San Diego, CA). R-spondin1-expressing 293T cells were a kind gift from Dr. Noah Shroyer (Baylor College of Medicine). Cultures were passaged every three to four days.

### Cytokine stimulation assays & gene target qPCR

Three to four days after last passage, 250 nM 4-hydroxytamoxifen (4OHT) was added to organoid cultures for 24 hours to allow A20 deletion. Mouse recombinant IL-17A, IFNγ (R&D Systems), or IFNα (PBL Assay Science, Piscataway, NJ) were added to cultures and incubated for 12 hours. Cells were harvested by adding PBS and pooling wells of the same condition into a single conical tube. Cultures were centrifuged, resuspended in Cell Recovery Solution (Corning), and incubated on a rotator at 4°C for 15 minutes. Cells were centrifuged, pellets were resuspended in Trizol and stored at -80°C, if for qPCR analysis, or snap frozen in a dry ice-ethanol bath and stored at -80°C, if for western blots. RNA was isolated using a phenol-chloroform extraction as above and cDNA generated by reverse transcriptase using the Omniscript RT kit (Qiagen). Transcripts of interest were probed for by qPCR using Taqman Gene Expression Assays (Thermofisher Scientific). All genes probed for are listed in S3 Table. Amplification reactions were performed on a StepOnePlus (Applied Biosystems). Cycle threshold (Ct) values were normalized to three housekeeping genes (*Rplp0*, *Gapdh*, *Actb*). The fold-change was calculated by normalizing to values for media alone, resulting in the control condition in each run to appear as 1.

### Immunoblotting

Cell lysates were made from snap frozen pellets of organoids using ice-cold 1% NP-40 (Calbiochem, San Diego, CA) containing 50mM Tris HCl pH 7.4, 150mM NaCl, and 10% glycerol and a protease inhibitor cocktail (cOmplete mini EDTA-free, Roche, Indianapolis, IN). Protein was quantified using a Pierce BCA Protein Assay Kit (Thermofisher Scientific) according to manufacturer’s instructions, with absorbance measured on a Spectramax M5 (Molecular Devices, Sunnyvale, CA) and analyzed using SoftmaxPro software (Molecular Devices). Lysates were combined with NuPAGE LDS Sample Buffer (Invitrogen, Carlsbad, CA) and NuPAGE Sample Reducing Agent (Invitrogen), and resolved on a NuPage precast Novex 4–12% Bis-Tris Protein Gel (Invitrogen) in volumes normalized to protein content alongside PageRuler Plus Prestained Ladder (Thermofisher Scientific). Protein was transferred onto a PVDF membrane. Nonspecific binding was blocked by incubation in 5% nonfat dry milk dissolved in Tris-Buffered Saline with Tween-20 (TBST) for one hour. Membrane was incubated in 5% Bovine Serum Albumin (BSA) TBST with 0.1% azide containing primary antibody at 4°C overnight on a shaker. Primary antibody for GAPDH was purchased from EMD Millipore (Billerica, MA), while all other primary antibodies used were purchased from Cell Signaling Technology (Danvers, MA). After washing with TBST, membrane was incubated with HRP-conjugated secondary polyclonal antibodies to mouse or rabbit IgGs (Cell Signaling Technology) in 5% milk TBST for one hour at room temperature. Signals were developed using SuperSignal West Pico, Dura, or Femto Chemiluminescent Substrate (Thermofisher Scientific). Blots were imaged on a ChemiDoc Touch Imaging System (Bio-Rad Laboratories). For reprobing, blots were stripped of existing antibodies by incubation in Restore Western Blot Stripping Buffer (Thermofisher Scientific) at 56°C with gentle rocking for 20–30 mins and incubated with primary and secondary antibodies as above. Band intensity was quantified using ImageLab Software (Bio-Rad Laboratories).

### Cytotoxicity assays in organoid cultures

Cultures were treated with 250 nM 4OHT for 24 hours prior to addition of cytokine. Organoids were stimulated with 10 ng/ml of IFNα (PBL Assay Sciences), IFNγ, or IL-17A (R&D Systems) for 24 hours and then stained with 1 μg/ml propidium iodide (PI) (Biolegend). Confocal imaging of intestinal organoids was performed on a Leica SP5 laser scanning confocal system (Leica Microsystems, Wetzlar, Germany) using a 10X dry objective. Images were acquired in a format of 512x512, with a line average of at least 3, scan speed of 400 Hz, and pinhole airy unit 1. Excitation for both PI and bright field was done with the 488nm laser line at 30% power with a detection band of 550-732nm. Image analysis was performed on the Leica Application Suite (Leica Microsystems). Cell death assays of intestinal organoids were performed by resuspending in Matrigel (Corning) and plating 25 μl per well in 96-well round bottom plates (Corning). After 24 hours, organoids were stimulated as indicated. Viability was measured using the CellTiter Glo 3D assay (Promega Corporation, Sunnyvale, CA) according to the manufacturer’s specifications. Luminescence was read on a SpectraMax M5 (Molecular Devices) and analyzed using SoftMax Pro (Molecular Devices).

### Treatment of HIV-infected individuals on ART with pegylated-IFNα2a

HIV-infected ART-treated participants of Clinical Trial NCT00594880 were enrolled based on pre-determined criteria, including viral load (<50 copies per ml as an indicator of effective suppression) and CD4 count (>450 cells per ul). Eligible individuals were treated with pegylated-IFNα2a (Roche), as published previously [54]. Cryopreserved PBMC samples taken from each participant at baseline and at five weeks post-initiation of IFNα administration were obtained for use in this study, providing paired samples from before and after cytokine treatment.

### Assessment of transcript levels in pegylated-IFNα2a-treated HIV-infected individuals

250 ng of RNA isolated from whole PBMCs were transcribed into cDNA using the SuperScript VILO cDNA Synthesis Kit (Invitrogen). Quantitative real-time PCR measuring *TNFAIP3* and *ISG15* using TaqMan real-time PCR was performed using the QuantStudio 6 Flex Real-Time PCR System (Applied Biosystems). Raw cycle threshold (Ct) numbers of amplified gene products were normalized to the housekeeping gene, *GAPDH*, to control for cDNA input amounts. Fold induction was determined using the comparative Ct method [79].

### Statistical analyses

For data obtained from clinical samples, Kruskal-Wallis tests were performed in Prism Version 6 (GraphPad, La Jolla, CA) (www.graphpad.com/scientific-software/prism/). A p-value of less than 0.05 (normal-based 95% Confidence Interval) was considered significant. Spearman correlations and associations were generated using R software (www.r-project.org) with general data analysis (https://cran.r-project.org/package=Hmisc) and statistical calculation (stats) packages. For data generated by murine organoid experiments, data were determined to be normally distributed and analyzed for significance by ANOVA in Stata (StataCrop LLC, College Station, Texas) (www.stata.com); post-hoc pairwise comparisons were made using t-tests with Scheffe adjustment for multiple comparisons. Analysis of qPCR measurements from PBMC samples taken from participants in the IFNα-treatment clinical trial was completed by paired t-test in Prism Version 6.

## Supporting information

S1 Fig Immunologic measurements of participants characterized by flow cytometry. (**A**) Percentages of gut CD4+ (blue) and CD8+ (teal) T cells in clinical subgroups. Black stars represent significance of differences in these subsets found in the clinical subgroups compared to uninfected controls. Red stars below error bars represent significance of differences of these subsets found in the ART-treated group compared to viremic untreated group. (**B**) Representative flow cytograms for each subgroup showing IL-17A and IL-22 producing gut CD4+ T cells after 5 hours of PMA/ionomycin stimulation. Quantification of (**C**) IL-17A and (**D**) IL-22 production in gut CD4+ T cells across all subjects. (**E**) Production of IL-17A by total gut lymphocytes, gated on live CD45+ events. (**F**) Immune activation, defined by CD38 and HLA-DR co-expression, on peripheral blood CD8+ T cells across subgroups. Statistical significance was determined by Kruskal-Wallis analysis. * P<0.05, **P<0.01, ***P<0.001, ****P<0.0001.
(EPS)

S2 Fig Differentially expressed genes between uninfected individuals and HIV-infected subgroups. (**A**) Percentages of CD45-EpCAM+ across participant subgroups. (**B**) Spearman correlation of A20 and *EPCAM* gene expression of all patients (uninfected in blue, viremic untreated in orange, HIV+ART+ in maroon) as assessed RNAseq. Pairwise comparison of IEC gene expression between uninfected controls and (**C**) ART-treated subjects or (**F**) viremic untreated subjects by RNAseq as analyzed by DESeq2 workflow. Genes of interest are highlighted in bold. (**D**) Spearman correlation of expression of indicated genes measured by RNAseq or qPCR in all subjects. (**E**) A20 mRNA levels in uninfected and viremic individuals, as well as HIV+ART+ participants subdivided by exclusion (-) or inclusion (+) of either (i) efavirenz, (ii) tenofovir, or (iii) abacavir in the treatment regimen.
(EPS)

S3 Fig Type I IFN signature in blood and gut of viremic subjects. (**A**) Ratio of kynurenine and tryptophan levels in plasma, assessed by liquid chromatography tandem mass spectrometry. (**B**) Expression of interferon stimulated genes *MX1* and *OAS2* in gut biopsies by RNAseq. Statistical significance was determined using a Kruskal-Wallis test. * P<0.05, **P<0.01.
(EPS)

S4 Fig HIV viremia is characterized by high levels of peripheral and gut-associated IFNγ. (**A**) IFNγ production across clinical subgroups. IFNγ cytokine levels in CD8+ T cells as measured by flow cytometry after a five hour stimulation with PMA/ionomycin. Statistical significance is determined by a Kruskal-Wallis test. (**B**) Transcript levels of IFNγ in whole rectosigmoid biopsies as assessed by qPCR and normalized to *ACTB* levels. Statistical significance is determined by Kruskal-Wallis. * P<0.05, **P<0.01.
(EPS)

S1 Table Statistical analyses of IFNγ-treated A20FL/FL villin-ERCre organoids. Analysis by ANOVA and post-hoc t-tests after adjustment for multiple comparison by Scheffe method of organoids treated with recombinant IFNγ. For each gene tested, p-value of the ANOVA test is listed above (orange). Gray indicates that the ANOVA yielded insignificance. p-values within the table represent significance after Scheffe adjustment for the indicated pairwise comparison.
(XLSX)

S2 Table Statistical analyses of IL-17-treated A20FL/FL villin-ERCre organoids. Analysis by ANOVA and post-hoc t-tests after adjustment for multiple comparison by Scheffe method of organoids treated with recombinant IL-17. For each gene tested, p-value of the ANOVA test is listed above (orange). Gray indicates that the ANOVA yielded insignificance. p-values within the table represent significance after Scheffe adjustment for the indicated pairwise comparison.
(XLSX)

S3 Table Table lists genes tested by qPCR in cytokine stimulation experiments in organoids. Magnitude of how A20 deletion affected IL-17 signaling was determined by calculating fold change of gene expression in the 4OHT- and cytokine co-treated conditions between Cre+ and Cre- organoids. Statistical significance of the difference between Cre+ and Cre- lines was determined by a t-test. Significant p-values are highlighted in green. For genes significantly altered by A20 deletion, upregulation is indicated in red.
(XLSX)
